# Efficacy of Pembrolizumab vs. Nivolumab Plus Ipilimumab in Metastatic NSCLC in Relation to PD-L1 and TMB Status

**DOI:** 10.3390/cancers16101825

**Published:** 2024-05-10

**Authors:** Walid Shalata, Natalie Maimon Rabinovich, Abed Agbarya, Alexander Yakobson, Yulia Dudnik, Ashraf Abu Jama, Ahron Yehonatan Cohen, Sondos Shalata, Ahmad Abu Hamed, Tahel Ilan Ber, Oshri Machluf, Gal Shoham Levin, Amichay Meirovitz

**Affiliations:** 1 The Legacy Heritage Cancer Center and Dr. Larry Norton Institute, Soroka Medical Center, Beer-Sheva 84105, Israel; 2 Medical School for International Health, Ben-Gurion University of the Negev, Beer-Sheva 84105, Israel; 3 Department of Oncology, Meir Medical Center, Kfar-Saba 44180, Israel; 4 Oncology Department, Bnai Zion Medical Center, Haifa 31048, Israel; 5 Nutrition Unit, Galilee Medical Center, Nahariya 22000, Israel; sondoss2@gmc.gov.il; 6 PhaseV Trials Ltd., Tel Aviv 67443, Israel

**Keywords:** non-small cell lung cancer (NSCLC), PDL-1, TMB, real-world data, immune checkpoint inhibitor, pembrolizumab, nivolumab, ipilimumab

## Abstract

**Simple Summary:**

This study focused on immune checkpoint inhibitor therapy effectiveness in patients with non-small cell lung cancer (NSCLC), particularly concerning programmed death ligand 1 (PD-L1) status and tumor mutational burden (TMB). The study utilized an Israeli multi-center registry spanning from January 2018 to December 2022, compiling detailed clinicopathological and molecular epidemiological data alongside treatment modalities. Clinical endpoints were assessed through Kaplan–Meier curves and Cox-regression models based on treatment assignment. While evidence on PD-L1 status and immune checkpoint inhibitors is established, data on TMB efficacy in both clinical trials and real-world settings are lacking. This study presents the largest academic real-world dataset on PD-L1 and TMB status in advanced NSCLC patients, suggesting comparable survival benefits despite limited direct comparisons.

**Abstract:**

The efficacy of immune checkpoint inhibitor (ICI) therapy concerning programmed death ligand 1 (PD-L1) status is well established in patients diagnosed with non-small cell lung cancer (NSCLC). However, there remains a paucity of evidence regarding the efficacy concerning tumor mutational burden (TMB) in both clinical trials and real-world data (RWD). In the current article, clinicopathological and molecular epidemiological data were meticulously collected, and treatment modalities were meticulously recorded. The final analysis included a study population of 194 patients. Median age was 67 years (range 37–86), with the majority being male (71.13%), and 85.71% of patients were either current or former smokers at diagnosis. Adenocarcinoma accounted for most diagnoses (71.65%), followed by squamous cell carcinoma (24.23%). In terms of PD-L1 status, 42.78% had an expression level below 1%, 28.35% had an expression between 1–49%, and 28.87% had an expression above 50%. The TMB ranged from 0 to 75, with a median of 10.31 (range 0–75) for PD-L1 expression below 1%, with a median of 9.73 (range 0.95–39.63) for PD-L1 expression between 1–49%, and a median of 9.72 (range 0.95–48) for PD-L1 expression above 50%. Corresponding to patients with low PDL-1 less than 1% and low TMB (0–5), the median overall survival (mOS) was 16 (*p* = 0.18), and 15 months (*p* = 0.22), patients with medium PDL-1 (1–49%) and medium TMB (5–10), the mOS was 15 (*p* = 0.18) and 16 months (*p* = 0.22), patients with high PDL-1 (>50) and high TMB (>10), the mOS was 24 (*p* = 0.18) and 21 (*p* = 0.22) months. This study represents the largest academic RWD dataset concerning PD-L1 and TMB status in patients with locally advanced and metastatic NSCLC.

## 1. Introduction

Lung cancer is a predominant contributor to global mortality, constituting approximately a fifth (20%) of all cancer-related deaths. Non-small cell lung cancer (NSCLC) comprises roughly 85% of lung cancer cases, despite advancements in early detection [1,2,3,4].

The 5-year survival rate of lung cancer in the United States has reached almost 20% in recent years. The prognosis of this illness hinges on the stage at which the diagnosis occurs. More than two-thirds of lung cancer sufferers are aged 65 or older, with only 3% being under 40. NSCLC is histologically categorized into subtypes: lung adenocarcinoma (LUAD); squamous cell carcinoma (SCC); large cell carcinoma; and adeno-squamous carcinoma. The challenging prognosis of NSCLC often results from 70% of patients being diagnosed at advanced disease stages. This leads to high rates of morbidity and mortality and a median survival period of approximately one year [5,6,7,8].

Identifying driver mutations is feasible through various molecular analysis methods. Advanced assays utilizing hybrid capture next-generation sequencing (NGS) facilitate the detection of a broad spectrum of common and rare mutations; however, their adoption is not universal. Instead, routine techniques such as fluorescence in situ hybridization (FISH), immunohistochemistry (IHC), Sanger sequencing, point-of-care testing, and laboratory-developed tests based on PCR technology are commonly employed [9,10,11,12,13,14].

Programmed death ligand 1 (PD-L1) is the sole biomarker validated for predicting overall survival advantage with immune checkpoint inhibitor (ICI) therapy. PD-L1 expression is assessed by IHC [6,7,8,9,10,11].

Over the past decade, ICIs have emerged and gained approval for treating NSCLC across various stages, including neo-adjuvant, adjuvant, locally advanced, and metastatic settings. ICI therapy functions by blocking the interaction between PD-L1 on tumor cells and programmed cell death protein 1 (PD-1) on T cells, empowering T cells to effectively target and eliminate tumor cells. Recent studies have evidenced that both ICI monotherapy and its combination with chemotherapy enhance survival outcomes compared to chemotherapy alone in patients with advanced NSCLC [8,9,10,11,12,13,14].

There have been multiple attempts to pinpoint various predictive biomarkers. Among the most intriguing and contentious is the tumor mutational burden (TMB). TMB denotes the count of mutations per megabase (Mut/Mb) of DNA sequenced within a specific cancer. A higher detection of mutations, and consequently an increase in neo-epitopes, enhances the likelihood that one or more neo-antigens could be immunogenic, triggering a T cell response [15,16,17,18,19,20,21].

Initially, TMB was earmarked as a biomarker for ICIs in melanoma, with subsequent studies hinting at a potential clinical role for TMB in NSCLC. However, initial data failed to be validated in a prospective study assessing overall survival (OS) as the primary endpoint [19,20,21,22,23].

The objective of the current study was to investigate whether the level of TMB influences the efficacy of immunotherapy (IO) in NSCLC patients.

## 2. Materials and Methods

### 2.1. Study Design

This is a retrospective, real-world, multi-center, observational study of patients receiving immunotherapy for locally advanced or metastatic NSCLC. Patient recruitment relied on electronic medical records, including all NSCLC patients who underwent treatment with pembrolizumab monotherapy or a combination of nivolumab with ipilimumab and chemotherapy from January 2018 to December 2022.

Patient data, including TMB levels, treatment regimens, metastasis location at diagnosis, response rates, PDL-1 value at diagnosis, and survival outcomes, were collected from medical records and databases. Statistical analyses, including regression models and survival analyses, were used to assess the impact of TMB levels on treatment response and overall survival. Subgroup analyses based on cancer type and treatment regimen were conducted to explore potential variations in treatment outcomes.

### 2.2. Study Population and Inclusion Criteria

Patients aged 18 years or older diagnosed with advanced or metastatic NSCLC were included in the study. They received ICI therapy (pembrolizumab or nivolumab with ipilimumab and chemotherapy) as first-line treatment following the standard of care during the study period, provided they tested negative for mutations in EGFR, ALK, MET, RET, BRAF, and ROS. Only patients with recorded progression-free survival (PFS) and overall survival (OS) were considered. Patients had Eastern Cooperative Oncology Group (ECOG) performance-status scores ranging from 0 to 4, indicating varying degrees of disability (Table 1). None of the patients had undergone previous ICI and/or systemic therapy for advanced or metastatic disease. They were either treated exclusively at Soroka Medical Center or had a comprehensive follow-up history in the center’s records. Upon admission to Soroka Medical Center’s Oncology Institute, each patient underwent evaluation and assessment by a multidisciplinary medical team comprising general medical and radiation oncologists, radiologists, pulmonologists, immunologists, nuclear physicians, thoracic surgeons, and pathologists. The treatment plan was tailored based on the patient’s condition, pathology, and imaging and was overseen by a primary physician assigned to manage the treatment course. Patients previously diagnosed with advanced or metastatic disease were primarily managed by medical oncologists, following National Comprehensive Cancer Network (NCCN) recommendations [24], with routine molecular profiling conducted whenever feasible.

### 2.3. Exclusion Criteria

Exclusion criteria included patients treated with chemotherapy only, cases with unknown (TMB) values, detection of tumor mutations in EGFR, ALK, ROS, RET, and BRAF, and patients diagnosed with two malignant primaries. These criteria were implemented to ensure the homogeneity and integrity of the study population, facilitating clearer analysis and interpretation of the research findings.

### 2.4. Treatment Administered

#### 2.4.1. Adenocarcinoma

Patients diagnosed with adenocarcinoma underwent a treatment protocol tailored by the attending physician. Each treatment cycle, spanning three weeks, was initiated with intravenous administration of cisplatin at a dosage of 75 mg per square meter of body-surface area or carboplatin adjusted based on the area under the concentration-time curve (AUC of 6–4), contingent on the patient’s performance status, alongside pemetrexed dosed at 500 mg per square meter of body-surface area. Additionally, nivolumab was administered intravenously at a dose of 360 mg every three weeks, and ipilimumab was given intravenously at a dose of 1 mg/kg every six weeks. After the initial two cycles of therapy, the platinum agent was discontinued, while pemetrexed, nivolumab, and ipilimumab were continued as maintenance therapy, following the regimen in the Checkmate 9LA trial [25]. Alternatively, pembrolizumab (in lieu of nivolumab and ipilimumab and after the initial four cycles of therapy; the platinum agent was discontinued) was administered intravenously at a dose of 200 mg every three weeks with pemetrexed, as in the Keynote 189 trial [26]. This treatment regimen was planned to be given for up to two years, or until the occurrence of unacceptable toxicity or disease progression. All patients received premedication for pemetrexed in accordance with institutional guidelines, including folic acid, vitamin B12, and glucocorticoids.

#### 2.4.2. Squamous Cell Carcinoma and Adeno-Squamous Cell Carcinoma

Patients diagnosed with squamous cell carcinoma and adeno-squamous cell carcinoma underwent a treatment protocol tailored by the attending physician. Each treatment cycle, spanning three weeks, was initiated with intravenous administration of cisplatin at a dosage of 75 mg per square meter of body-surface area or carboplatin adjusted based on the area under the concentration–time curve (AUC of 4–6), contingent on the patient’s performance status, along with paclitaxel at 175 mg per square meter of body-surface area. Additionally, nivolumab was administered intravenously at a dose of 360 mg every three weeks, and ipilimumab was given intravenously at a dose of 1 mg/kg every six weeks. After the initial two cycles of therapy, the chemotherapy (paclitaxel and platinum) agent was discontinued, while nivolumab, and ipilimumab were continued as maintenance immunotherapy, following the regimen in the Checkmate 9LA trial [25]. Alternatively, pembrolizumab (in lieu of nivolumab and ipilimumab and after the initial four cycles of therapy; the platinum and paclitaxel agents were discontinued) was administered intravenously at a dose of 200 mg every three weeks, as in the Keynote 407 trial [27]. This treatment regimen was planned to be given for up to two years, or until the occurrence of unacceptable toxicity or disease progression.

### 2.5. Data and Statistical Analysis

Descriptive statistics were calculated for all parameters in the study, including percentages, ranges, means, medians, and interquartile ranges (IQRs). The log rank test was utilized to compute OS and PFS, with significance set at *p* < 0.05. Data was presented as frequencies (n) and percentages (%) or medians and ranges. A confidence interval (CI) of 95% and *p*-values were calculated. The statistical analysis was conducted by PhaseV Trials Ltd.

The study parameters comprised both numerical data (e.g., PFS, OS in months) and binary data (e.g., presence of adverse events, Yes/No or 1/0). Descriptive statistics summarized treatment outcome measures, including adverse events (AEs), employing median (m), OS, range, frequencies (n), PFS, and percentages.

Kaplan–Meier analysis was utilized to assess overall survival rates in the study population. The analysis included stratification of the study population into subgroups based on TMB levels (lower tertile, middle tertile, upper tertile); TMB levels and type of immunotherapy received; PDL1 levels (<1%, 1–49%, ≥50%); PDL1 levels and type of immunotherapy received. Analyses that included treatment stratification were conducted separately for each type of immunotherapy to avoid biased results due to the non-random nature of the treatment assignment in the study population. The multivariate log-rank test was used to test the hypothesis of a survival difference between the subgroups within each stratified analysis.

In addition, a multivariate Cox proportional hazards (PH) model was used to analyze the association between TMB, PD-L1, the type of immunotherapy received, and overall survival. The model included main effect terms for TMB (numerical), PDL1 (binary: high vs. low\medium, 50% was used as cutoff), the treatment (ipilimumab-nivolumab vs. pembrolizumab), and the two covariate-by-treatment interaction terms (TMB*treatment and PDL1 level*treatment). It is important to note that the analysis did not account for the non-random treatment assignment and therefore was not designed to uncover a causal relationship between the choice of treatment and the OS. Consequently, the coefficient of the treatment term should not be interpreted as indicating a causal effect but rather as reflecting the association present under the current assignment mechanism.

## 3. Results

One hundred ninety-four patients met the eligibility criteria. Detailed patient information is shown in Table 1. The median age of the study participants was 67 years, with ages ranging from 37 to 86 years. Gender distribution revealed 138 males, constituting approximately 71.13% of the population, with 56 females accounting for 28.87% of the cohort. Smoking status at diagnosis indicated that 109 individuals (55.61%) were current smokers, 59 (30.1%) were former smokers, and 26 (13.27%) had never smoked. ECOG status distribution showed 35 participants with a score of 0 (20.12%), 107 with a score of 1 (61.49%), and 52 with a score of 2 (29.89%). Histologically, adenocarcinoma appeared as the predominant type, with 139 cases (71.65%), followed by squamous cell carcinoma (SCC) with 47 cases (24.23%). Co-mutations were observed, with KRAS being the most prevalent (55 cases, 28.35%), followed by TP53 (52 cases, 26.8%), and STK11 (18 cases, 9.28%), among others. PD-L1 expression levels were categorized as <1% in 83 cases (42.78%), and the median TMB was calculated as 10.31 mutations per megabase. PD-L1 expression levels of 1–49% occurred in 55 cases (28.35%), and the median TMB was calculated as 9.73 mutations per megabase. PD-L1 expression levels of >50% occurred in 56 cases (28.87%), and the median TMB was calculated as 9.72 mutations per megabase. The TMB range was from 0 to 75 per megabase. Metastasis locations were varied, with the most common being lung metastases observed in 113 cases (58.25%), followed by lymph node involvement in 100 cases (51.55%). Other locations included bone (67 cases, 34.54%), pleural effusion (43 cases, 22.16%), brain (35 cases, 18.04%), adrenal (30 cases, 15.46%), liver (25 cases, 12.89%), pericardial effusion (4 cases, 2.06%), and spleen (1 case, 0.52%).

In terms of PD-L1 status, the study revealed notable differences in median overall survival across different expression levels. For patients with PD-L1 expression levels below 1%, the median overall survival was recorded at 16 months, with a range spanning from 3 to 46 months.

**Figure 1:**
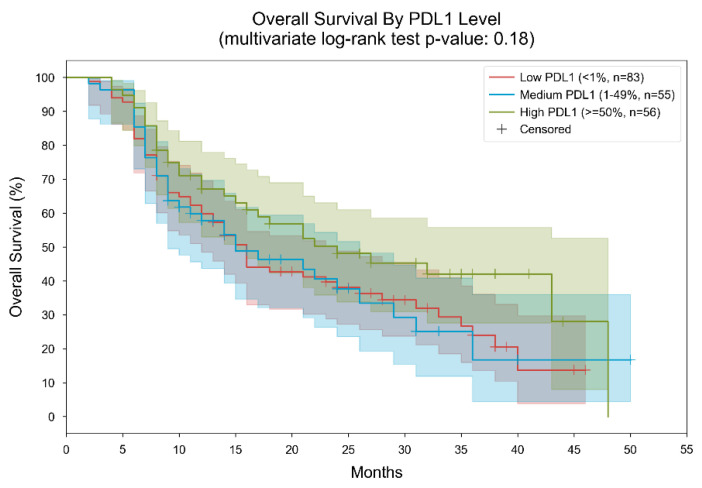
Kaplan–Meier curves for overall survival, stratified by PDL1 level. Patients were divided into 3 subgroups based on their measured PDL1 level (low: “<1%”, medium: “1–49%”, high: “≥50%”). In the low PDL1 group, the median overall survival was 16 months; in the medium PDL1 group, the median overall survival was 15 months; In the high PDL1 group, the median overall survival was 24 months. The *p*-value of the multivariate log-rank statistic for survival difference between the three groups was 0.18. In a Cox proportional hazards (PH) model with terms for PDL1 level, the low PDL1 level was associated with a hazard ratio (HR) of 1.47 (95% CI [0.95, 2.28], *p* = 0.09) compared to the high PDL1 level; the medium PDL1 level was associated with an HR of 1.46 (95% CI [0.9, 2.38], *p* = 0.12) compared to the high PDL1 level.

Conversely, patients exhibiting PD-L1 expression levels between 1% and 49% experienced a slightly shorter median overall survival of 15 months, ranging from 2 to 50 months. Individuals with PD-L1 expression exceeding 50% demonstrated the highest median overall survival of 24 months, with a range extending from 4 to 48 months (*p*-value 0.18) (Figure 1).

**Figure 2:**
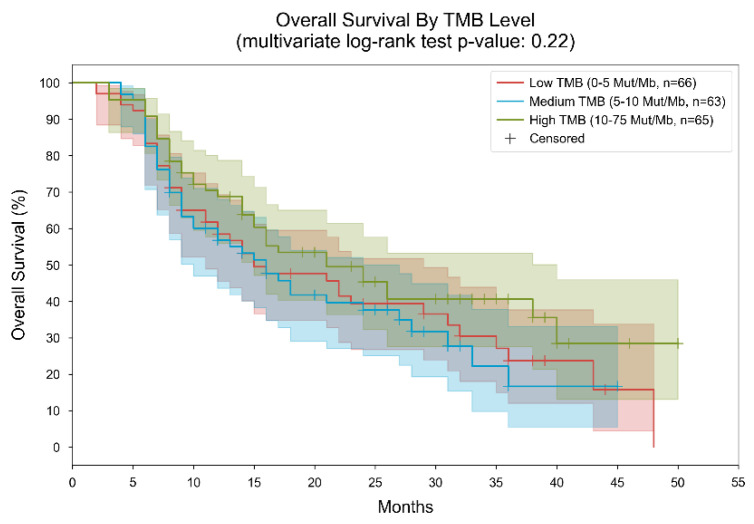
Kaplan–Meier curves for overall survival, stratified by TMB level. Patients were divided into 3 subgroups based on their measured TMB level (low: “0–5” mut/MB, medium: “5–10” mut/Mb, high: “10–75” mut/Mb). In the low TMB group, the median overall survival was 15 months; in the medium TMB group, the median overall survival was 16 months; in the high TMB group, the median overall survival was 21 months. The *p*-value of the multivariate log-rank statistic for survival difference between the three groups was 0.22. In a Cox proportional hazards (PH) model with terms for TMB levels, the low TMB level was associated with a hazard ratio (HR) of 1.36 (95% CI [0.88, 2.1], *p* = 0.17) compared to the high TMB level; the medium TMB level was associated with an HR of 1.45 (95% CI [0.93, 2.26], *p* = 0.1) compared to the high TMB level.

Regarding TMB status, distinct patterns in median overall survival were observed across different mutation levels. Among patients with a TMB not exceeding 5 mutations per megabase, the median overall survival was recorded at 15 months, spanning from 2 to 48 months (Figure 2).

In cases where the TMB ranged between 5 and 10 mutations per megabase, the median overall survival increased slightly to 16 months, with a narrower range of 4 to 45 months. Conversely, individuals with a TMB surpassing 10 mutations per megabase exhibited the longest median overall survival of 21 months, with a range from 3 to 50 months (*p*-value 0.22).

**Figure 3:**
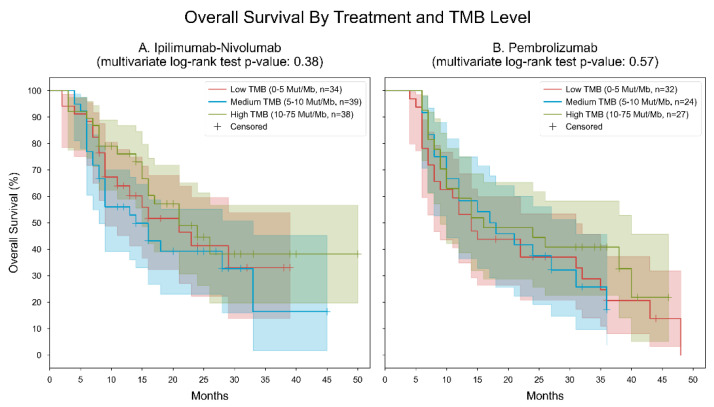
Kaplan–Meier curves for overall survival, stratified by treatment and TMB level. Patients were divided into 6 subgroups based on their measured TMB level (low: “0–5” mut/MB, medium: “5–10” mut/Mb, high: “10–75” mut/Mb) and the type of immunotherapy they received (ipilimumab-nivolumab or pembrolizumab). Two separate analyses were performed, one for patients with ipilimumab-nivolumab and the other for patients with Pembrolizumab. Cohort (**A**). Ipilimumab-Nivolumab. In the low TMB group, the median overall survival was 21 months; in the medium TMB group, the median overall survival was 14 months; in the high TMB group, the median overall survival was 21 months. The *p*-value of the multivariate log-rank statistic for survival difference between the three groups was 0.38. In a Cox proportional hazards (PH) model for patients treated with ipilimumab-nivolumab, which included terms for TMB levels, the low TMB level was associated with a hazard ratio (HR) of 1.23 (95% CI [0.64, 2.31], *p* = 0.53) compared to the high TMB level; the medium TMB level was associated with an HR of 1.54 (95% CI [0.84, 2.82], *p* = 0.16) compared to the high TMB level.; Cohort (**B**). Pembrolizumab. In the low TMB group, the median overall survival was 14 months; in the medium TMB group, the median overall survival was 17 months; in the high TMB group, the median overall survival was 16 months. The *p*-value of the multivariate log-rank statistic for survival difference between the three groups was 0.57. In a Cox proportional hazards (PH) model for patients treated with pembrolizumab, which included terms for TMB levels, the low TMB level was associated with a hazard ratio (HR) of 1.37 (95% CI [0.74, 2.51], *p* = 0.31) compared to the high TMB level; the medium TMB level was associated with an HR of 1.31 (95% CI [0.67, 2.53], *p* = 0.43) compared to the high TMB level.

The analysis of two cohorts comparing the efficacy of ipilimumab-nivolumab and pembrolizumab across different TMB groups revealed distinct patterns in median OS and statistical significance. In the cohort treated with ipilimumab-nivolumab, there were varying OS rates across TMB groups, with the low and high TMB groups showing a similar median OS of 21 months, while the medium TMB group exhibited a notably lower median OS of 14 months. Despite these differences, the multivariate log-rank statistic yielded a *p*-value of 0.38, suggesting no statistically significant divergence in survival outcomes among the TMB groups. Conversely, in the pembrolizumab cohort, efficacy demonstrated more nuanced results. While the low TMB group showed a median OS of 14 months, the medium TMB group displayed a modest increase to 17 months, followed by a slight decline in the high TMB group to 16 months. The corresponding multivariate log-rank statistic produced a *p*-value of 0.57, indicating an even weaker level of statistical significance and suggesting a lack of discernible survival differences between TMB groups under pembrolizumab treatment (Figure 3).

**Figure 4:**
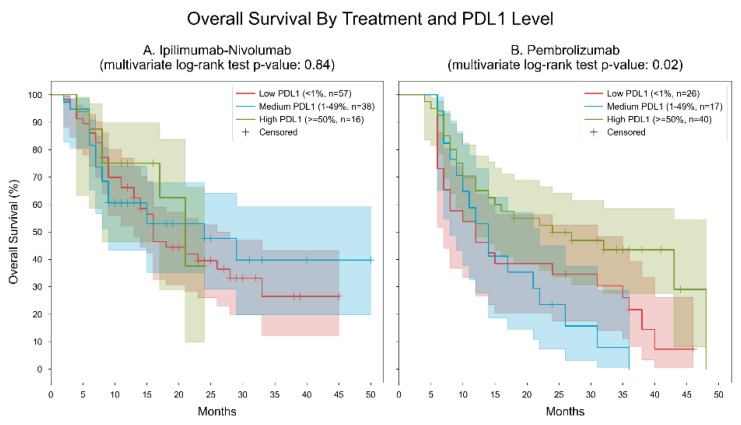
Kaplan–Meier curves for overall survival, stratified by treatment and PDL1 level. Patients were divided into 6 subgroups based on their measured PDL1 level (low: “<1%”, medium: “1–49%”, high: “≥50%”) and the type of immunotherapy they received (ipilimumab-nivolumab or pembrolizumab). Two separate analyses were performed, one for patients with ipilimumab-nivolumab and the other for patients with pembrolizumab. Cohort (**A**). Ipilimumab-nivolumab. In the low PDL1 group, the median overall survival was 16 months; in the medium PDL1 group, the median overall survival was 24 months; in the high PDL1 group, the median overall survival was 21 months. The *p*-value of the multivariate log-rank statistic for survival difference between the three groups was 0.84. In a Cox proportional hazards (PH) model for patients treated with ipilimumab-nivolumab, which included terms for PDL1 levels, the low PDL1 level was associated with a hazard ratio (HR) of 1.24 (95% CI [0.55, 2.8], *p* = 0.61) compared to the high PDL1 level; the medium PDL1 level was associated with an HR of 1.11 (95% CI [0.47, 2.66], *p* = 0.81) compared to the high PDL1 level.; Cohort (**B**). Pembrolizumab. In the low PDL1 group, the median overall survival was 12 months; in the medium PDL1 group, the median overall survival was 14 months; in the high PDL1 group, the median overall survival was 24 months. The *p*-value of the multivariate log-rank statistic for survival difference between the three groups was 0.02. In a Cox proportional hazards (PH) model for patients treated with pembrolizumab, which included terms for PDL1 levels, the low PDL1 level was associated with a hazard ratio (HR) of 1.87 (95% CI [1.04, 3.36], *p* = 0.036) compared to the high PDL1 level; the medium PDL1 level was associated with an HR of 2.3 (95% CI [1.2, 4.42], *p* = 0.012) compared to the high PDL1 level.

Two separate analyses were conducted to assess the efficacy of ipilimumab-nivolumab and pembrolizumab across varying levels of programmed death-ligand 1 (PD-L1) expression. In Cohort A, which focused on Ipilimumab-Nivolumab, the median OS differed among PD-L1 groups, with the medium PD-L1 group demonstrating the highest median OS of 24 months, followed by the high PD-L1 group with 21 months, and the low PD-L1 group with 16 months. Despite these variations, the multivariate log-rank statistic returned a non-significant *p*-value of 0.84, indicating no significant discrepancy in survival outcomes between the PD-L1 groups. Conversely, Cohort B, which examined Pembrolizumab, revealed notable distinctions in median OS across PD-L1 groups. Specifically, the low PD-L1 group exhibited the lowest median OS of 12 months, followed by the medium PD-L1 group with 14 months, while the high PD-L1 group displayed the highest median OS of 24 months. Importantly, the multivariate log-rank statistic yielded a significant *p*-value of 0.02, underscoring a significant difference in survival outcomes among the different PD-L1 groups receiving pembrolizumab treatment (Figure 4).

**Figure 5:**
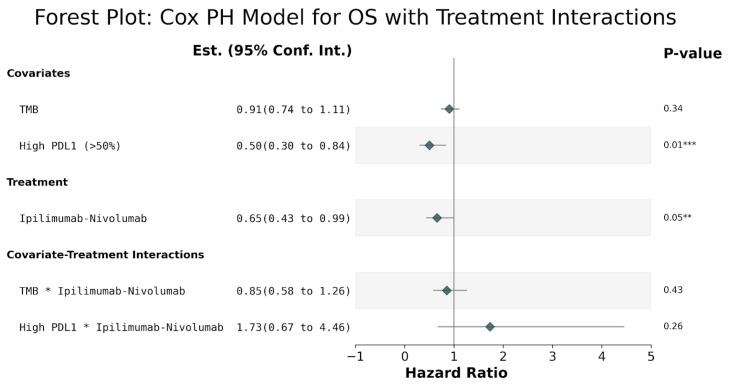
Results from a Cox proportional hazards model for overall survival with terms for TMB (numerical), PDL1 (binary, high vs. low\medium), the treatment (ipilimumab-nivolumab vs. pembrolizumab), and the two covariate-by-treatment interactions. *** High PDL1 was associated with reduced hazard compared to low-medium PDL1 (HR = 0.5, 95% CI [0.3, 0.84], *p* = 0.01). ** Ipilimumab-nivolumab treatment was associated with reduced hazard compared to pembrolizumab treatment (HR = 0.65, 95% CI [0.43, 0.99], *p* = 0.05). * The terms for TMB and for the treatment-by-covariate interactions were not statistically significant.

The findings from the treatment interactions analysis, which encompassed variables such as tumor mutational burden (TMB), programmed death-ligand 1 (PD-L1) expression (classified as high vs. low/medium), and treatment regimen (ipilimumab-nivolumab vs. pembrolizumab), along with their interactions, revealed noteworthy correlations. Specifically, high PD-L1 expression was associated with a decreased hazard compared to low-medium PDL1 (log(HR) = −0.69, 95% CI (−1.2, −0.17), *p* = 0.01). Additionally, patients administered ipilimumab-nivolumab demonstrated a reduced hazard relative to those receiving pembrolizumab treatment (log(HR) = −0.69, 95% CI (−1.2, −0.17), *p* = 0.01). Additionally, patients administered ipilimumab-nivolumab demonstrated a reduced hazard relative to those receiving pembrolizumab treatment (log(HR) = −0.42, 95% CI (−0.84, −0.01), *p* = 0.05); (nevertheless, statistical significance was not attained for terms related to TMB or treatment-covariate interactions), (Figure 5).

**Figure 6:**
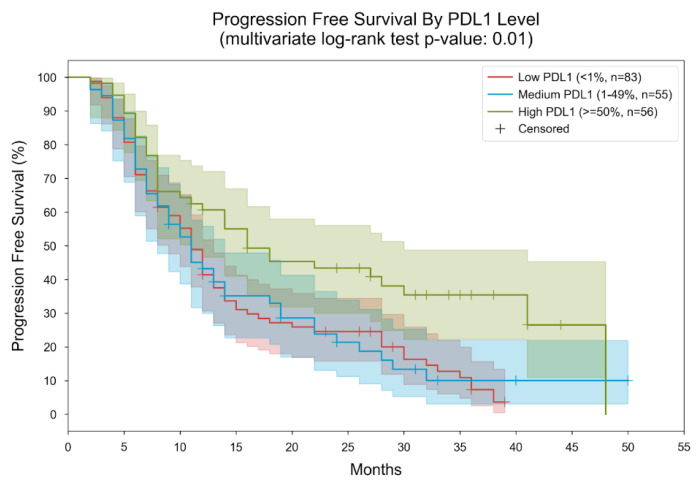
Kaplan–Meier curves for PFS, stratified by PDL1 level. Patients were divided into three subgroups based on their measured PDL1 level (low: “<1%”, medium: “1–49%”, high: “≥50%”). In the low PDL1 group, the median PFS was 11 months; in the medium PDL1 group, the median PFS was 11 months; in the high PDL1 group, the median PFS was 16 months. The *p*-value of the multivariate log-rank statistic for PFS difference between the three groups was 0.01. In a Cox proportional hazards (PH) model with terms for PDL1 level, the low PDL1 level was associated with a hazard ratio (HR) of 1.85 (95% CI [1.23, 2.77], *p* = 0.003) compared to the high PDL1 level; the medium PDL1 level was associated with an HR of 1.79 (95% CI [1.15, 2.78], *p* = 0.01) compared to the high PDL1 level.

In the analysis of PFS by PDL1 level, patients were categorized into three subgroups based on their measured PDL1 levels: low (<1%), medium (1–49%), and high (≥50%). Within these groups, the median PFS was found to be 11 months for both the low and medium PDL1 groups, while it extended to 16 months for the high PDL1 group (Figure 6).

**Figure 7:**
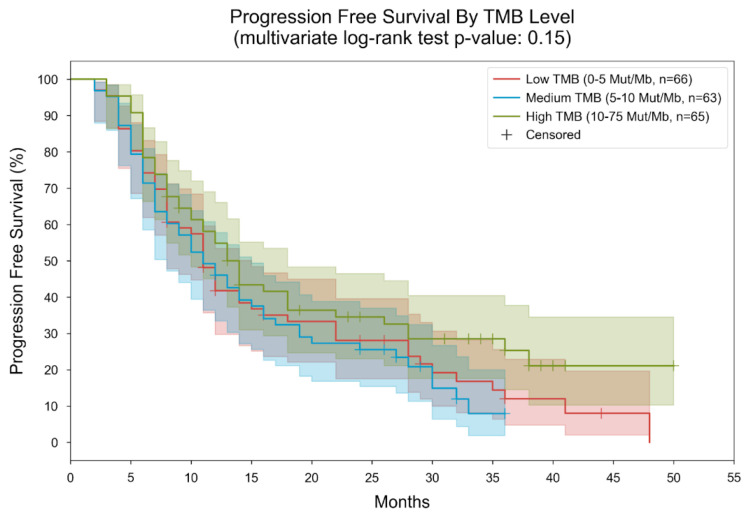
Kaplan–Meier curves for progression-free survival, stratified by TMB level. Patients were divided into 3 subgroups based on their measured TMB level (low: “0–5” mut/MB, medium: “5–10” mut/Mb, high: “10–75” mut/Mb). In the low TMB group, the median PFS was 11 months; in the medium TMB group, the median PFS was 11 months; in the high TMB group, the median PFS was 14 months. The *p*-value of the multivariate log-rank statistic for PFS difference between the three groups was 0.15. In a Cox proportional hazards (PH) model with terms for TMB levels, the low TMB level was associated with a hazard ratio (HR) of 1.35 (95% CI [0.91, 2.0], *p* = 0.13) compared to the high TMB level; the medium TMB level was associated with an HR of 1.44 (95% CI [0.97, 2.15], *p* = 0.07) compared to the high TMB level.

Patients were stratified into three subgroups based on their measured tumor mutational burden (TMB) level: low (0–5 mutations per megabase [mut/MB]), medium (5–10 mut/MB), and high (10–75 mut/MB). Within these groups, the median overall survival was found to be 11 months for both the low and medium TMB groups, while it extended to 14 months for the high TMB group. The multivariate log-rank statistic yielded a *p*-value of 0.15, indicating no significant survival difference among the three groups (Figure 7).

Regarding the PFS, patients were categorized into subgroups based on their measured PDL1 levels (<1% for low, 1–49% for medium, and ≥50% for high) and the type of immunotherapy administered (ipilimumab-nivolumab or pembrolizumab). Two distinct analyses were conducted, one for patients receiving ipilimumab-nivolumab and another for those receiving pembrolizumab.

**Figure 8:**
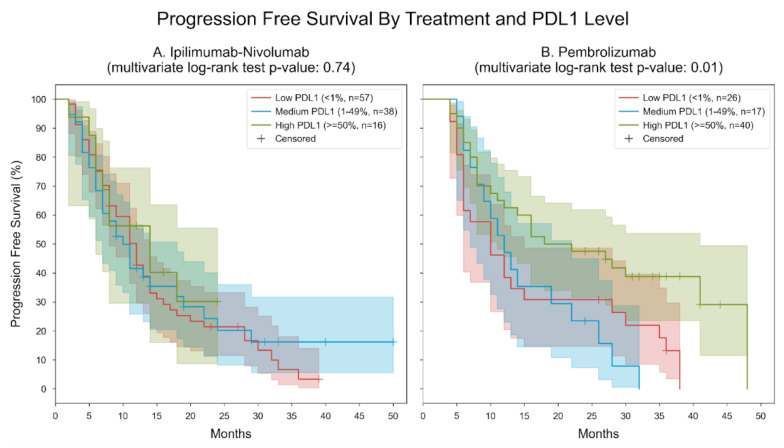
Kaplan–Meier curves for PFS, stratified by treatment and PDL1 level. Patients were divided into 3 subgroups based on their measured PDL1 level (low: “<1%”, medium: “1–49%”, high: “≥50%”) and the type of immunotherapy they received (ipilimumab-nivolumab or pembrolizumab). Two separate analyses were performed, one for patients with ipilimumab-nivolumab and the other for patients with pembrolizumab. Panel (**A**). Ipilimumab-nivolumab. In the low PDL1 group, the median PFS was 12 months; in the medium PDL1 group, the median PFS was 10 months; in the high PDL1 group, the median PFS was 14 months. The *p*-value of the multivariate log-rank statistic for PFS difference between the three groups was 0.74. In a Cox proportional hazards (PH) model for patients treated with ipilimumab-nivolumab, which included terms for PDL1 levels, the low PDL1 level was associated with a hazard ratio (HR) of 1.3 (95% CI [0.66, 2.58], *p* = 0.45) compared to the high PDL1 level; the medium PDL1 level was associated with an HR of 1.22 (95% CI [0.59, 2.52], *p* = 0.59) compared to the high PDL1 level. Panel (**B**). Pembrolizumab. In the low PDL1 group, the median PFS was 10 months; in the medium PDL1 group, the median PFS was 12 months; in the high PDL1 group, the median PFS was 18 months. The *p*-value of the multivariate log-rank statistic for PFS difference between the three groups was 0.01. In a Cox proportional hazards (PH) model for patients treated with pembrolizumab, which included terms for PDL1 levels, the low PDL1 level was associated with a hazard ratio (HR) of 2.06 (95% CI [1.16, 3.65], *p* = 0.014) compared to the high PDL1 level; the medium PDL1 level was associated with an HR of 2.2 (95% CI [1.16, 4.2], *p* = 0.017) compared to the high PDL1 level.

Concerning ipilimumab-nivolumab treatment, the median PFS was observed to be 12 months for the low PDL1 group, 10 months for the medium PDL1 group, and 14 months for the high PDL1 group. The multivariate log-rank statistic for PFS difference among the three groups yielded a *p*-value of 0.74. Focusing on pembrolizumab treatment, the median PFS was 10 months for the low PDL1 group, 12 months for the medium PDL1 group, and 18 months for the high PDL1 group. The multivariate log-rank statistic for PFS difference among the three groups yielded a *p*-value of 0.01 (Figure 8).

PFS was analyzed and stratified by treatment and TMB level. Patients were categorized into six subgroups based on their measured TMB level (low: 0–5 mutations per megabase [mut/MB], medium: 5–10 mut/MB, high: 10–75 mut/MB) and the type of immunotherapy they received (ipilimumab-nivolumab or pembrolizumab), leading to two distinct analyses.

**Figure 9:**
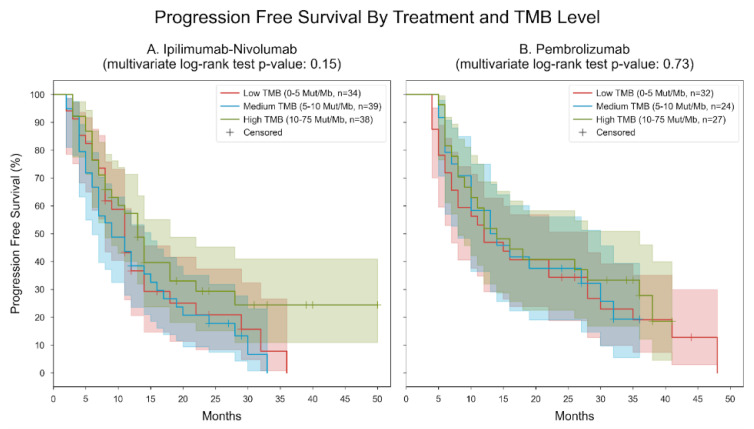
Kaplan–Meier curves for progression-free survival, stratified by treatment and TMB level. Patients were divided into 6 subgroups based on their measured TMB level (low: “0–5” mut/MB, medium: “5–10” mut/Mb, high: “10–75” mut/Mb) and the type of immunotherapy they received (ipilimumab-nivolumab or pembrolizumab). Two separate analyses were performed, one for patients with ipilimumab-nivolumab and the other for patients with pembrolizumab. Panel A. Ipilimumab-nivolumab. In the low TMB group, the median PFS was 11 months; in the medium TMB group, the median PFS was 9 months; in the high TMB group, the median PFS was 13 months. The *p*-value of the multivariate log-rank statistic for PFS difference between the three groups was 0.15. In a Cox proportional hazards (PH) model for patients treated with ipilimumab-nivolumab, which included terms for TMB levels, the low TMB level was associated with a hazard ratio (HR) of 1.45 (95% CI [0.85, 2.48], *p* = 0.18) compared to the high TMB level; the medium TMB level was associated with an HR of 1.62 (95% CI [0.97, 2.71], *p* = 0.07) compared to the high TMB level. Panel B. Pembrolizumab. In the low TMB group, the median PFS was 12 months; in the medium TMB group, the median PFS was 13 months; in the high TMB group, the median PFS was 14 months. The *p*-value of the multivariate log-rank statistic for PFS difference between the three groups was 0.73. In a Cox proportional hazards (PH) model for patients treated with pembrolizumab, which included terms for TMB levels, the low TMB level was associated with a hazard ratio (HR) of 1.27 (95% CI [0.71, 2.27], *p* = 0.43) compared to the high TMB level; the medium TMB level was associated with an HR of 1.16 (95% CI [0.61, 2.2], *p* = 0.65) compared to the high TMB level.

For patients treated with ipilimumab-nivolumab, in the low TMB group, the median PFS was 11 months, while it was 9 months for the medium TMB group and 13 months for the high TMB group. The multivariate log-rank statistic for PFS difference among the three groups yielded a *p*-value of 0.15. In contrast, for patients treated with pembrolizumab, in the low TMB group, the median PFS was 12 months; in the medium TMB group, it was 13 months; and in the high TMB group, it extended to 14 months. The multivariate log-rank statistic for PFS difference among the three groups yielded a *p*-value of 0.73 (Figure 9).

Adverse events (AEs) were categorized by type and severity (Grade 1–2). For nivolumab plus ipilimumab: The most prevalent AE was anemia, with a total occurrence of 129 cases (66.49% of total cases), followed by fatigue with 80 cases (41.24%), and hypothyroidism with 41 cases (21.13%). Other common AEs included rash (15.98%), diarrhea (11.86%), and pruritus (11.86%). For pembrolizumab, anemia was also the most frequently reported AE, with 59 cases (30.41%), followed by fatigue with 63 cases (32.47%), and hypothyroidism with 24 cases (12.37%). Rash, diarrhea, and pruritus were among the common AEs reported, but at lower frequencies compared to nivolumab plus ipilimumab. Furthermore, certain AEs, such as hyperthyroidism, were observed exclusively in the nivolumab plus ipilimumab group, while others, such as neutropenia and vomiting, were relatively rare across both treatment groups. For AEs greater than Grade 3, nausea exhibited the highest frequency, with a total of 5 occurrences reported, constituting 2.58% of the total cases. Within this category, 3 occurrences (1.55%) were associated with ipilimumab and nivolumab, while 2 (1.03%) were linked to pembrolizumab. Neutropenia followed, with 3 occurrences (1.55%), of which 2 (1.03%) were attributed to ipilimumab and nivolumab and 1 (0.52%) to pembrolizumab. Abdominal pain, vomiting, and hepatitis each had 2 occurrences (1.03%), all of which were associated with ipilimumab and Nivolumab. Moreover, diarrhea and pneumonitis were reported with 2 (1.03%) and 1 (0.52%) occurrences, respectively, with varying attribution between the two treatment groups. Additional Grade >3 AEs included myositis, neuropathy, myocarditis, polyneuropathy, periorbital edema, and death, each reported with a single occurrence and distinct attribution to the treatment regimens (Table 2).

## 4. Discussion

Lung cancer remains the foremost contributor to cancer-related mortality among patients globally, underscoring the critical need for early detection and tailored treatment strategies to enhance patient outcomes, particularly in cases of NSCLC [28,29,30]. Designing treatment plans for NSCLC patients hinges upon molecular profiling and PDL1 testing, guiding the administration of receptor monoclonal antibodies (mAb) or small-molecule TKIs tailored to the specific driver mutation [29,30]. Although the management of EGFR- and ALK-mutated NSCLC is firmly established, investigations into treatment approaches for other mutations, particularly those less common, are ongoing. TMB can be evaluated through next-generation sequencing techniques. TMB is broadly defined as the count of somatic mutations per megabase of genomic sequence under scrutiny. TMB plays a crucial role in generating immunogenic neopeptides showcased on major histocompatibility complexes on the tumor cell surface, thereby influencing patient response to ICIs. These tumor-specific neoantigens stem from somatic mutations and can significantly contribute to tumor-specific T cell-mediated anti-tumor immunity following checkpoint signal inhibition. Beyond neoantigen quantity, emerging evidence suggests that quality is also paramount, with high-quality neoantigens potentially encompassing expressed clonal neoantigens in vital genes. Such neoantigens possess the ability to bind to multiple loci in heterozygous alleles and resist repression or deletion due to their genomic position. Mounting evidence suggests that TMB might serve as a predictive biomarker for tumor response to ICIs across various cancer types [9,10,11,12,13].

In a study involving patients with previously treated, unresectable, or metastatic solid tumors (KEYNOTE 158), a TMB-high status (≥10 mut/Mb) correlated with a clinically significant enhancement in the effectiveness of the anti-PD-1 antibody pembrolizumab. Notably, responses were observed across various tumor types, and a microsatellite instability-high status did not solely explain the increased clinical benefits within the TMB-high subgroup. Consequently, based on these findings, the FDA sanctioned pembrolizumab monotherapy for the subset of solid-tumor patients exhibiting TMB ≥10 mut/Mb, particularly those resistant to treatment and lacking satisfactory alternative options.

While insights from KEYNOTE 158 underscore the significance of TMB in patient selection for cancer therapy, critical issues persist, including the determination and implementation of a fixed TMB cutoff based on pan-cancer data. Notably, variations in TMB distributions across different cancer types hold relevance in establishing optimal TMB thresholds to facilitate its utility as a predictive biomarker for immunotherapy [2]. These findings underscore the potential prognostic implications of both PD-L1 expression levels and TMB in predicting overall survival outcomes among the studied population. Such insights are crucial for informing personalized treatment strategies and guiding clinical decision-making in the management of relevant conditions.

Our analysis, when compared to the findings from the Checkmate 9LA trial [25], underscores the effectiveness of both types of immunotherapies in treating lung cancer. By stratifying our data according to tumor PD-L1 expression levels, we observed distinct median overall survival (OS) durations. For patients with low PD-L1 expression (<1%), the median OS stood at 16 months, whereas those with medium PD-L1 expression (1–49%) exhibited a median OS of 15 months. Remarkably, individuals with high PD-L1 expression (≥50%) displayed a significantly longer median OS of 24 months. Contrasting these results with those from the KEYNOTE 189 trial [26], which focused on non-squamous NSCLC, the overall study population reported a median OS of 19.4 months. However, when analyzed according to PD-L1 expression, individuals with high PD-L1 achieved an outstanding median OS of 29.6 months, while the medium PD-L1 and low PD-L1 subgroups reported median OS durations of 19.8 and 9.6 months, respectively. Similarly, in the KEYNOTE 407 trial [27] targeting squamous NSCLC, the overall study population demonstrated a median OS of 17.1 months. Among patients with different PD-L1 expression levels, those with medium PD-L1 exhibited the longest median OS at 18.9 months, followed by low PD-L1 and high PD-L1 groups, both with a median OS of 15 months. In the analysis of various subgroups, specifically examining the relationship between PD-L1 and TMB alongside outcomes or survival as a third dimension, no significant findings emerged, except for the significance observed in the PD-L1 values under treatment with ipilimumab plus nivolumab across the overall population, as noted in Figure 5.

The meta-analysis conducted on TMB in patients treated with pembrolizumab across the KEYNOTE-189 and KEYNOTE-407 trials yielded significant insights into OS and PFS [31]. However, despite thorough examination, the analysis did not reveal a notable association between TMB values (above or below 10 mutations per exome) and the efficacy of pembrolizumab plus chemotherapy. This result was further supported by our study’s findings, which also showed no statistically significant differences in TMB values within the pembrolizumab-treated cohort. Additionally, the OS benefit observed with ipilimumab and nivolumab plus chemotherapy compared to chemotherapy alone showed no significant difference between TMB ≥10 and <10 mut/Mb, as well as between TMB ≥16 and <16 mut/Mb subgroups. However, there was a trend towards higher OS benefit in the TMB ≥20 versus <20 mut/Mb subgroups. In terms of PFS and objective response rate (ORR), the magnitude of benefit was notably higher in TMB-high compared to TMB-low patients. These findings suggest that higher TMB levels are associated with enhanced PFS and ORR benefits with ipilimumab and nivolumab plus chemotherapy compared to chemotherapy alone [32]. Such observations in our study were not statistically significant.

When comparing the median progression-free survival (mPFS) results reported in the main trials with our own study, KEYNOTE-189 (pembrolizumab) reported an mPFS of 9.0 months [26], while KEYNOTE-407 (pembrolizumab) reported 6.4 months [27]. In contrast, our study revealed a mPFS of 10 months, and the 9LA study (Ipilimumab-Nivolumab) showed an mPFS of 6.7 months [25], whereas ours stood at 12 months.

These disparities between our study and the original trials regarding mPFS could be attributed to the limitations of real-world retrospective data collection. Assessing mPFS in such trials is challenging due to intra-observer variation in radiologic response evaluation, especially when considering a patient’s clinical status. Therefore, the higher mPFS observed in our study may better reflect the actual treatment duration, rather than a strictly objective mPFS, considering factors such as the treating physician’s intention to maximize treatment time using this combination and the less structured nature of radiologic evaluation.

Regarding comparison with other real-world data and due to the geographic restriction of our study to Israeli centers, we encountered another research investigation focused on whether TMB values correlate with efficacy and outcomes in patients with NSCLC treated with nivolumab plus ipilimumab. Notably, this study involved patients from the USA. Interestingly, the findings from this study mirrored ours, revealing that elevated levels of tumor mutation burden are linked to the enhanced efficacy of combination immunotherapy [33]. These results suggest that TMB’s association with treatment efficacy might not be significantly influenced by geographical location.

It was previously reported that when exploring immunotherapy outcomes for NSCLC patients concerning TMB, the consensus was that elevated TMB levels are analogous to high levels of PD-L1, yielding improved results and heightened responsiveness to immunotherapy. The articles emphasized that as the number of neoantigens within a tumor rises, so does the likelihood of inciting a T-cell response to one or more of these neoantigens. Additionally, it underscored a robust correlation between the quantity of candidate neoantigens per tumor and tumor mutational burden (TMB) [34,35].

Focusing on subgroups that were previously examined, in correlations between TMB and various factors. It was observed that TMB tends to be higher in men compared to women and that squamous histology in lung carcinoma exhibits higher TMB levels compared to adenocarcinoma. Additionally, smoking status was explored, with indications that tobacco use may influence TMB due to the accumulation of somatic mutations caused by carcinogens in tobacco smoke, resulting in a higher neoantigen load. This potentially leads to an enhanced immunotherapy response in patients with a history of smoking [36,37,38,39]. Interestingly, our study yielded similar results, with a median TMB value of 10.38 (range 0–75) in males and 8.98 (range 0–31.73) in females. Regarding smoking history, patients who never smoked exhibited lower median TMB values (5.99, range 0–16.5) compared to patients with a history of smoking (past or present), who had a median TMB of 10.59 (range 0–75). but mOS was not significantly influenced by the TMB in correlation with smoking status, in which smokers had an mOS of 17.73 months and non-smokers 17.69 months.

However, it is noteworthy that mOS did not show significant differences based on TMB in correlation with smoking status. Both smokers and non-smokers exhibited similar mOS, with smokers having an mOS of 17.73 months and non-smokers having an mOS of 17.69 months.

Overall, treatment-related adverse events (TRAEs) were reported in 97% of patients in the nivolumab plus ipilimumab arm, whereas in the pembrolizumab arm, TRAEs were reported in 89% of patients. Notable differences in AE occurrences between this current study and the clinical trials were noted. Overall, TRAEs were more prevalent in the nivolumab plus ipilimumab arm of our trial compared to the clinical trial (Checkmate 9LA) (98% vs. 92%) [25]. Conversely, in the pembrolizumab arm, the overall TRAEs were lower in our study (94% vs. 99.8% in Keynote 189 and 98.2% in Keynote 407) [26,27]. However, we noted disparities in the occurrence rates of certain AEs compared to those reported in clinical trials. For instance, in our study, the incidence of anemia was higher in the nivolumab plus ipilimumab arm (66.49%) compared to the clinical trial (24%) (Checkmate 9LA) [25]. For pembrolizumab therapy, we observed an anemia rate of 30.41%, compared to 46.2% in Keynote 407 and 53.2% in Keynote 189 [26,27]. Similarly, the prevalence of fatigue in the nivolumab plus ipilimumab arm was 41.24% in our study, notably higher than the reported 17% in the clinical trial (Checkmate 9LA) [25]. For pembrolizumab therapy, the fatigue incidence was 32.47% in our study, compared to 22.7% in Keynote 407 and 40.7% in Keynote 189. Moreover, we reported fewer grade 3 TRAEs (9.77%) compared to the nivolumab plus ipilimumab arm in the clinical trial (48%) (Checkmate 9LA) [25]. For pembrolizumab therapy, the incidence of TRAEs was reported as 96.8% in Keynote 407 and 67.2% in Keynote 189, which differed from our findings [26,27]. In our study, TRAEs tended to be milder when compared to the findings from the CheckMate 9LA trial 9250, Keynote 407 [27], and Keynote 189 trials [26]. This trend is likely a result of the extensive clinical experience of our treatment team and their heightened vigilance in monitoring patients, which has been honed through the increasing adoption of immunotherapy and chemo-immunotherapy in real-world practice. Prompt identification and effective management of these adverse events not only benefited patients but also potentially enhanced overall treatment outcomes.

In comparison to other real-world data, a systematic review conducted a head-to-head comparison between nivolumab plus ipilimumab and pembrolizumab as first-line chemotherapy-free treatments for PD-L1-positive non-small cell lung cancer [40]. The review found that although indirect comparisons suggested that nivolumab plus ipilimumab may offer a longer PFS compared to pembrolizumab, this difference did not reach statistical significance in our study. Additionally, nivolumab plus ipilimumab did not demonstrate superiority over pembrolizumab in terms of OS, consistent with our study findings.

Moreover, our study observed a higher incidence of both all-grades and ≥grade 3 treatment-related adverse events with nivolumab plus ipilimumab compared to pembrolizumab, aligning with the findings reported in the systematic review.

The disparity between that systematic review study and our own lies in our observation that TMB could potentially act as a prognostic indicator for treatment response, akin to PDL-1 values. Despite this association not attaining statistical significance, our analysis, as illustrated in the previous graphs, indicates its possible clinical significance.

In our investigation of biomarker combinations, our study emphasizes PD-L1 and TMB status as individual biomarkers. Future research suggestions could include exploring the potential synergistic effects of combining these biomarkers or incorporating additional molecular markers to refine patient selection for specific treatments. Also, we suggest more prospective studies or randomized controlled trials, which could strengthen the evidence base for the efficacy of the compared treatments in specific patient subgroups.

The limitations of our study stem from several factors. Firstly, the reliance on data sourced from only three institutions may limit the generalizability of our findings. Additionally, being a retrospective study, there are discrepancies in the follow-up period compared to the original study, which necessitates a comprehensive assessment of various endpoints. Furthermore, the accuracy of performance status designation may be questionable. In a real-world setting, where the primary goal of treating physicians is to optimize first-line treatment duration using a combination of immunotherapy and chemotherapy, a pragmatic efficacy endpoint such as “time-to-treatment discontinuation” may hold significance. In these scenarios, radiologic evaluations may lack standardization, and ECOG status monitoring may be less rigorous, potentially leading to inaccuracies in recorded data. Our study underscores the importance of meticulously documenting patient information, including ECOG scores and other relevant data, as this could significantly impact future research and studies. Additionally, it is essential to acknowledge that not all TRAEs may be accurately reported in electronic patient records. Ensuring accurate and comprehensive documentation is crucial for advancing medical knowledge and ultimately improving patient care. Finally, we have intentionally chosen not to compare squamous and non-squamous diagnoses in our study. Since the treatment is not randomly assigned (acknowledging its retrospective design), a direct comparison of the survival curves of the two treatments may lead to erroneous conclusions because the selection of cases was arbitrary, completely depending on the treating physician. For example, the apparent superiority of one treatment over the other may be the result of assigning patients in a better condition to that treatment rather than a causal effect. This decision aims to mitigate potential confusion in the results across varied cases, ensuring the reliability of our findings and avoiding inaccuracies or misunderstandings among healthcare professionals.

## 5. Conclusions

By assessing the performance of immune checkpoint inhibitor drugs in relation to PD-L1 expression and TMB levels, this study aims to provide clinicians with valuable information to guide treatment decisions for metastatic NSCLC patients. These results can help in identifying patient subgroups who may optimally benefit from specific treatment regimens based on personalized biomarker profiles, which may help reduce unnecessary TRAEs of immunotherapies.

## Figures and Tables

**Table 1:** Baseline disease characteristics of the study population (*n* = 194).

		Median (Range)
**Age (years)**		67 (37–86)
	Male	67 (37–86)
	Female	66 (40–82)
**Gender**		**Frequencies (percentage-%)**
	Male	138 (71.13)
	Female	56 (28.87)
**Smoking status** (at diagnosis)		
	Current	109 (55.61)
	Former	59 (30.1)
	Never	26 (13.27)
**ECOG status**		
	0	35 (20.12)
	1	107 (61.49)
	2	52 (29.89)
**Histology**		
	Adenocarcinoma	139 (71.65)
	SCC	47 (24.23)
	Adenosquamous cell carcinoma	5 (2.58)
	Others *	3 (1.55)
**Immunotherapy administered**		
	nivolumab plus ipilimumab	111 (57.22)
	pembrolizumab	83 (42.78)
**Co-mutations**		
	KRAS	55 (28.35)
	TP53	52 (26.8)
	STK11	18 (9.28)
	CDK 4–6	8 (4.12)
	KEAP1	6 (3.09)
	CDKN2A	6 (3.09)
	EGFR amplification	5 (2.58)
	BRAF non V600E	5 (2.58)
**PD-L1 status**	**Frequencies (percentage-%)**	**TMB Median (range)**
PD-L1 expression < 1%	83 (42.78)	10.31 (0–75)
PD-L1 expression 1–49%	55 (28.35)	9.73 (0.95–39.63)
PD-L1 expression >50%	56 (28.87)	9.72 (0.95–48)
**Metastasis locations**		
	Lung	113 (58.25)
	Lymph nodes involvement	100 (51.55)
	Bone	67 (34.54)
	Pleural effusion	43 (22.16)
	Brain	35 (18.04)
	Adrenal	30 (15.46)
	Liver	25 (12.89)
	Pericardial effusion	4 (2.06)
	Spleen	1 (0.52)

Abbreviations: ECOG, Eastern Cooperative Oncology Group; PD-L1, programmed cell death-ligand 1; SCC, squamous cell carcinoma; KRAS, Kirsten rat sarcoma virus.; TP 53, tumor protein p53; STK11, serine/threonine kinase 11; CDK, cyclin-dependent kinase; EGFR, Epidermal growth factor receptor; KEAP1, Kelch-like ECH-associated protein 1; TMB, tumor mutational burden and BRAF, v-raf murine sarcoma viral oncogene homolog B1. Note: Immunohistochemistry was performed on tissue biopsy samples. The tumor’s PD-L1 expression was measured using Ventana’s XT Benchmark (Roche Diagnostics, Basel, Switzerland) using IHC PharmDx (clone 22C3, Dako), UltraView detection kit (FDA approved, Ventana). * Giant cell carcinoma and Pleomorphic carcinoma.

**Table 2:** Treatment-related adverse events of nivolumab plus ipilimumab or pembrolizumab.

**Type of AE (Grade 1–2)**	**Total Occurrences**	**Keytruda**	**Ipilimumab and Nivolumab**	**P, 95% CI (Difference in Proportions)**
Anemia	188 (97.42%)	59 (30.41%)	129 (66.49%)	<0.001, (0.2377, 0.4073)
Fatigue	143 (73.71%)	63 (32.47%)	80 (41.24%)	0.066, (0.0023, 0.3112)
Hypothyroidism	65 (33.51%)	24 (12.37%)	41 (21.13%)	0.008, (0.0484, 0.4136)
Rash	44 (22.68%)	13 (6.70%)	31 (15.98%)	0.013, (0.0451, 0.4032)
Diarrhea	35 (18.04%)	12 (6.19%)	23 (11.86%)	0.056, (0.0058, 0.3007)
Pruritus	33 (17.01%)	10 (5.15%)	23 (11.86%)	0.017, (0.0554, 0.3946)
Arthralgia	24 (12.37%)	9 (4.64%)	15 (7.73%)	0.199, (0.0109, 0.3609)
Transaminases	24 (12.37%)	6 (3.09%)	18 (9.28%)	0.174, (0.0375, 0.4135)
Pneumonitis	21 (10.82%)	6 (3.09%)	15 (7.73%)	0.197, (0.0284, 0.3748)
Hyperthyroidism	10 (5.15%)	0	10 (5.15%)	0.003, (0.1069, 0.6531)
Creatinine elevation	8 (4.12%)	2 (1.03%)	6 (3.09%)	<0.001, (0.2377, 0.4073)
**Type of AE (Grade ≥ 3)**	**Total Occurrences**	**Keytruda**	**Ipilimumab and Nivolumab**	**P, 95% CI (Difference in Proportions)**
Nausea	5 (2.58%)	2 (1.03%)	3 (1.55%)	0.564, (0.3456, 0.6456)
Neutropenia	3 (1.55%)	1 (0.52%)	2 (1.03%)	0.564, (0.3456, 0.6456)
Abdominal pain	2 (1.03%)	0	2 (1.03%)	0.564, (0.3456, 0.6456)
Vomiting	2 (1.03%)	0	2 (1.03%)	0.564, (0.3456, 0.6456)
Hepatitis	2 (1.03%)	0	2 (1.03%)	0.564, (0.3456, 0.6456)
Diarrhea	2 (1.03%)	1 (0.52%)	1 (0.52%)	1, (0.9616, 0.9616)
Pneumonitis	1 (0.52%)	0	1 (0.52%)	1, (0.9616, 0.9616)
Myositis	2 (1.03%)	2 (1.03%)	0	0.564, (0.3456, 0.6456)
Neuropathy	1 (0.52%)	1 (0.52%)	0	1, (0.9616, 0.9616)
Myocarditis	1 (0.52%)	1 (0.52%)	0	1, (0.9616, 0.9616)
Polyneuropathy	1 (0.52%)	1 (0.52%)	0	1, (0.9616, 0.9616)
Periorbital edema	1 (0.52%)	1 (0.52%)	0	1, (0.9616, 0.9616)
Death	1 (0.52%)	0	1 (0.52%)	NA

Abbreviation: AE, adverse events; CI, confidence interval; NA, not applicable, Note- the results were calculated by the chi-square tests and 95% confidence intervals, P, *p*-value.

## Data Availability

The data either resides within the article itself or can be obtained from the authors upon making a reasonable request.
